# Disrupting KATP channels diminishes the estrogen-mediated protection in female mutant mice during ischemia-reperfusion

**DOI:** 10.1186/1559-0275-11-19

**Published:** 2014-05-06

**Authors:** Jianjiong Gao, Dong Xu, Grzegorz Sabat, Hector Valdivia, Wei Xu, Nian-Qing Shi

**Affiliations:** 1 Computational Biology Center and Center for Molecular Oncology, Memorial Sloan-Kettering Cancer Center, New York, NY 10065, USA; 2 Department of Computer Science and CS Bond Life Sciences Center, University of Missouri, Columbia, MO 65211, USA; 3 Biotechnology Center, Mass Spectrometry Facility, University of Wisconsin, Madison, WI 53706, USA; 4 Department of Internal Medicine, University of Michigan, 2800 Plymouth Ave., 26-235 N, Ann Arbor, MI 48105, USA; 5 McArdle Laboratory for Cancer Research, University of Wisconsin, 1400 University Ave., Madison, WI 53706, USA; 6 Department of Medicine, University of Wisconsin, Room 8418, WIMR II, 1111 Highland Ave., Madison, WI 53705, USA

**Keywords:** KATP channel, Sulfonylurea receptor, Myocardial infarction, Gender difference, Estrogen, Estrogen receptor, Glycosylation

## Abstract

**Background:**

Estrogen has been shown to mediate protection in female hearts against ischemia-reperfusion (I-R) stress. Composed by a Kir6.2 pore and an SUR2 regulatory subunit, cardiac ATP-sensitive potassium channels (KATP) remain quiescent under normal physiological conditions but they are activated by stress stimuli to confer protection to the heart. It remains unclear whether KATP is a regulatory target of estrogen in the female-specific I-R signaling pathway. In this study, we aimed at delineating the molecular mechanism underlying estrogen modulation on KATP channel activity during I-R.

**Materials and methods:**

We employed KATP knockout mice in which SUR2 is disrupted (SUR2KO) to characterize their I-R response using an *in vivo* occlusion model. To test the protective effects of estrogen, female mice were ovariectomized and implanted with 17β-estradiol (E2) or placebo pellets (0.1 μg/g/day, 21-day release) before receiving an I-R treatment. Comparative proteomic analyses were performed to assess pathway-level alterations between KO-IR and WT-IR hearts.

**Results and discussion:**

Echocardiographic results indicated that KO females were pre-disposed to cardiac dysfunction at baseline. The mutant mice were more susceptible to I-R stress by having bigger infarcts (46%) than WT controls (31%). The observation was confirmed using ovariectomized mice implanted with E2 or placebo. However, the estrogen-mediated protection was diminished in KO hearts. Expression studies showed that the SUR2 protein level, but not RNA level, was up-regulated in WT-IR mice relative to untreated controls possibly via PTMs. Our antibodies detected different glycosylated SUR2 receptor species after the PNGase F treatment, suggesting that SUR2 could be modified by N-glycosylation. We subsequently showed that E2 could further induce the formation of complex-glycosylated SUR2. Additional time-point experiments revealed that I-R hearts had increased levels of N-glycosylated SUR2; and DPM1, the first committed step enzyme in the N-glycosylation pathway. Comparative proteomic profiling identified 41 differentially altered protein hits between KO-IR and WT-IR mice encompassing those related to estrogen biosynthesis.

**Conclusions:**

Our findings suggest that KATP is likely a downstream regulatory target of estrogen and it is indispensable in female I-R signaling. Increasing SUR2 expression by N-glycosylation mediated by estrogen may be effective to enhance KATP channel subunit expression in I-R.

## Background

Myocardial infarction (MI) is a life-threatening event that can cause sudden cardiac arrest in patients; and those who survive the first MI likely have repeated incidences and develop heart failure [1]. During the on-set of MI, cardiac cells die within minutes from insufficient blood supply and oxygen, leading to irreversible injuries to the myocardium. Existing epidemiological data reveals that pre-menopausal women have a relatively lower risk of MI than age-matched men [2,3]. For example, MI incidences occurred in females are only 1/3 of their male counterparts in the 35–44 and 45–54 years-old groups [4]. However, this “female advantage” diminishes upon aging as estrogen level declines, supporting the notion that estrogen is a key modulator in mediating protection to a female heart [2,3]. Estrogen exerts its effects by binding to estrogen receptors (ER), ERα and ERβ, to regulate the downstream targets [5]. Both ER subtypes are detected in various cardiac cellular compartments encompassing nucleus, plasma membrane and mitochondria [6,7]. These receptors are likely integral members of the female cardioprotective network but they may govern different signal transduction pathways [8,9].

ATP-sensitive potassium channels (KATP) are known to play a pivotal role in conferring protection to the heart [10]. These channels remain closed under normal physiological conditions but they open in response to cellular stress such as ischemia. The opening of KATP channels is thought to re-polarize cardiac cell membrane and reduce calcium loading to the heart [11]. Sarcolemmal KATP channels primarily contain a Kir6.2 pore [12] and a sulfonylurea receptor 2 (SUR2) regulatory subunit [13-15], where SUR2 regulates the pore activity and channel kinetics. More recent studies have shown that KATP density in plasma membrane is significantly higher in female than male cardiomyocytes [16], suggesting that female hearts may possess higher KATP activity. Administrating KATP blockers to both genders of mice before an ischemic insult readily diminishes the female advantage against ischemia-reperfusion (I-R) stress [17]. In aged female myocytes, however, KATP channel density dramatically declines but remains unchanged in aged male cells [18]. These observations have provided a basis for estrogen modulation on KATP activity but the underlying mechanism is not fully understood.

In this report, we characterized the I-R response in SUR2 knockout (SUR2KO) female mice using a combined approach of ovariectomized models and comparative proteomics. Our findings identified KATP as a downstream regulatory target of estrogen and it plays a critical role in the mechanistic pathway of female stress signaling.

## Results and discussion

### SUR2KO female mice display cardiac dysfunction at baseline

**Figure 1:**
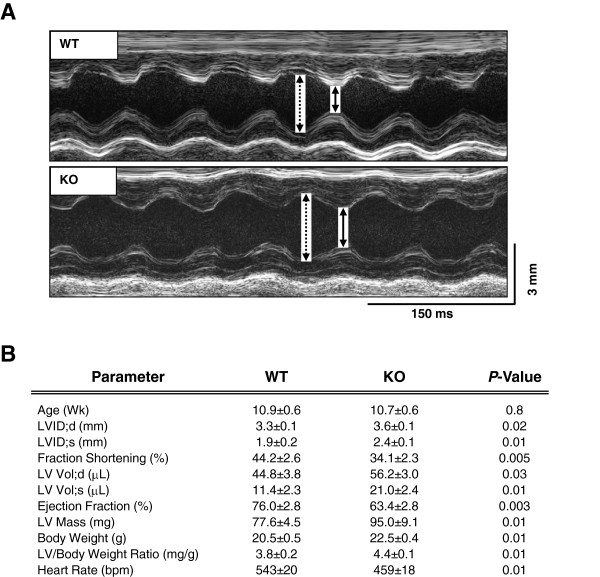
**Cardiac performance in SUR2KO and WT intact mice under basal conditions. (A)** Recorded echocardiographic images. Dashed line: left ventricular (LV) chamber dimension in diastole; solid line: LV chamber dimension in systole. **(B)** Summary data on LV function parameters in SUR2KO (n = 10) and WT (n = 8) mice. *P*-values are shown in the figure.

SUR2KO mice were previously generated by inserting a disruption cassette into exons12-16 of SUR2 [19]. Our prior study has shown that the SUR2 long forms are disrupted in KO hearts [20,21]. In this study, cardiac performance was evaluated in KO and WT female mice using echocardiography (Figure 1A). Fractional shortening or ejection fraction was significantly lowered in KO hearts relative to WT controls (Figure 1B). KO mice also exhibited markedly enlarged left ventricles (LV) with slower heart rates. Our results suggested that KO females were pre-disposed to cardiac dysfunction under basal conditions.

### SUR2KO females have larger infarcts post ischemia-reperfusion

**Figure 2:**
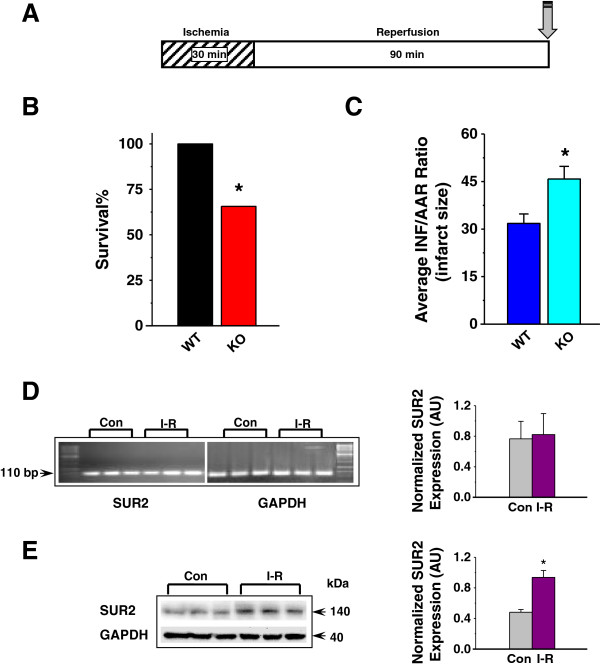
**Ischemia**-**reperfusion (I-R) response in SUR2KO and WT mice. (A)** Treatment protocol. Grey arrows indicate endpoints when mouse hearts were harvested. **(B)** Survival% from KO and WT mice post IR. **(C)** Average infarct sizes recorded from IR-treated hearts. Infarcts are calculated as a ratio of infarcted area (INF) over area-at-risk (AAR). n = 5-7. *: *p* < 0.05 KO vs WT. AAR/LV ratios are shown in the supplement Additional file 1: Figure S1. **(D)** Quantitative RT-PCR of SUR2 transcripts in untreated (Con) and I-R hearts of WT mice. The signals were normalized to GAPDH. **(E)** Representative Western blots in mouse LV samples. Anti-SUR2 (BNJ2) was used as the primary antibody (1:1000). Secondary antibody was added at 1:10,000. The blots were stripped and hybridized with GAPDH (1:2000), and the density values were normalized to GAPDH (in arbitrary unit). In **D-E**, n = 4, *: *p* < 0.05.

An *in vivo* occlusion model [22,23] was employed to assess I-R response in SUR2KO and WT mice. During the course of developing our protocols to evaluate the KO performance, various reperfusion lengths (1.5, 2, 4 or 24 h) were tested. KO female mice, however, experienced an unexpected high mortality rate when >2 h reperfusion was used. Our optimized protocol thus included a 30-min ischemia phase followed by 90-min reperfusion (Figure 2A). Even with the shortened reperfusion length, 44% of KO females did not survive the procedure, indicating that they were more susceptible to I-R stress. Compared to WT-IR hearts (31%, n = 8), KO-IR hearts (46%, n = 8) displayed significantly larger infarcts, indicating that KO females experienced worse cardiac damage (Figure 2B). This finding differs from our earlier data in KO males. Those mice were found to be “constitutively” protected from I-R stress by having smaller infarcts than WT controls in two different I-R model studies [21,24]. The results suggest that SUR2 long form-based KATP channels are indispensable in female protection but they may not be required in conferring protection to a male heart. The different I-R responses detected in both genders of SUR2KO mice revealed that KATP may be an important component that contributes to gender-specific difference in cardioprotection.To determine whether SUR2 expression was altered in I-R, RNA or protein was isolated from LV tissues excised from untreated or I-R hearts isolated from WT female mice. qRT-PCR study did not detect any significant differences in SUR2 transcripts between the two groups of mice (Figure 2C). However, SUR2 protein level was significantly increased 2-fold in the I-R hearts relative to the untreated controls (Figure 2D). The data indicated that the increased SUR2 protein level might be due to post-translational modifications (PTMs).

### The protective effect of estrogen is diminished in SUR2KO females

**Figure 3:**
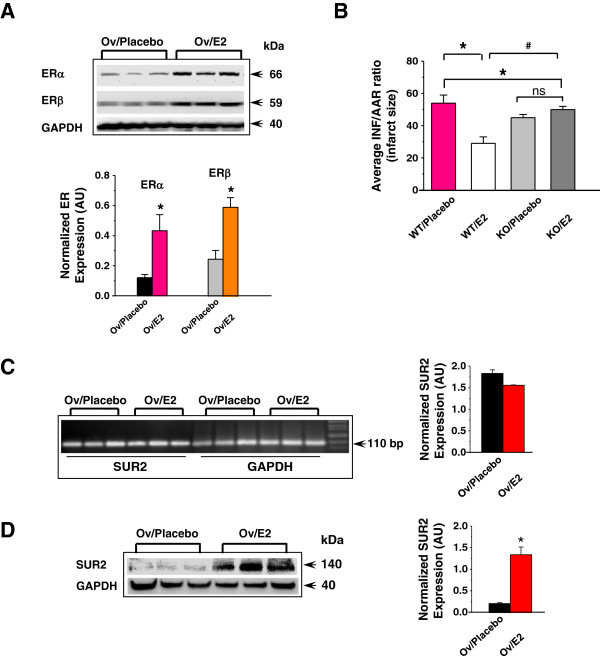
**Estrogenic effects in ovariectomized mouse models. (A)** Representative Western blots of ER expression post I-R. **(B)** Average infarct sizes (INF/AAR) recorded from ovariectomized mice implanted with E2 or placebo pellets. n = 4-6. *: *p* < 0.05 placebo vs E2; ^#^: *p* < 0.05 KO vs WT. AAR/LV ratios are shown in the supplement Additional file 1: Figure S1. **(C)** Quantitative RT-PCR of SUR2 transcripts in ovariectomized WT mice implanted with E2 or placebo pellets. The signals were normalized to GAPDH. **(D)** Representative Western blots in ovariectomized mice implanted with E2 or placebo pellets post I-R. Anti-SUR2 (BNJ-2) was used as the primary antibody (1:1000). Secondary antibody was added at 1:10,000. The blot was stripped and hybridized with GAPDH (1:2000), and the density values were normalized to GAPDH.

The estrogenic effect was subsequently evaluated in SUR2KO and WT female mice using ovariectomized models. Ovariectomized mice that were implanted with either 17β-estradiol (E2) or placebo pellets were used (0.1 μg/g/day, 21-day release). To ensure that the E2 delivery was effective, protein expression of both estrogen receptors were measured before the end of E2 releasing in both WT-E2 and WT-placebo mice. Protein levels of ERα and ERβ were significantly higher in WT-E2 hearts relative to WT-placebo controls (Figure 3A), suggesting that the delivery was satisfactory. These mice were then subjected to our I-R protocol to assess infarct size. Average infarcts (Figure 3B-C and Additional file 1) from WT-placebo hearts (57%, n = 4) were significantly larger than WT-E2 hearts (29%, n = 5), consistent with previous reports on the protective effect of estrogen [2] and our intact mouse model study (Figure 2B-C). Conversely, no significant differences in infarct size were found between KO-placebo (43%, n = 4) and KO-E2 mice (50%, n = 6), indicating that the protective effect of estrogen was abolished.

qRT-PCR data detected no significant differences in SUR2 transcripts between WT-E2 and WT-placebo hearts post I-R (Figure 3D). Western blot results showed that WT-E2 mice had a 6-fold higher SUR2 level than WT-placebo mice (Figure 3E). The data suggested that the increased SUR2 protein expression might occur at the post-translational level, which was mediated by estrogen. This observation agreed with a previous report showing that E2-ERβ action can induce S-nitrosylation, a PTM mechanism, to up-regulate levels of a subset of proteins with protective properties [25].

### SUR2 is modified by N-glycosylation

**Figure 4:**
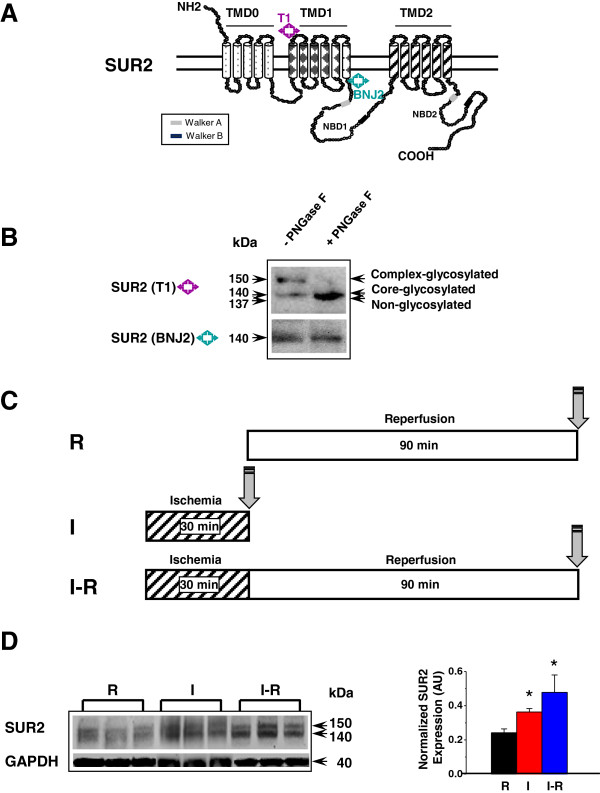
**SUR2 is a complex**-**glycosylated protein. (A)** SUR2 topology and epitope positions for T1 and BNJ2. **(B)** De-glycosylation of SUR2 protein by PNGase F. T1 (1:2000) or BNJ-2 (1:1000) was used as the primary antibody. BNJ-2 was used as a negative control for T1 and a loading control. Secondary antibody was added at 1:10,000. **(C)** Treatment protocols for R, I and I-R. Grey arrows indicate endpoints when mouse hearts were harvested. **(D)** Representative Western blots using R-, I- or IR- treated WT LV samples. T1 was used as the primary antibody (1:2000). Secondary antibody was added at 1:10,000. n = 4. *, *p* < 0.05. The blot was stripped and re-probed with GAPDH (1:2000), and the density values were normalized to GAPDH.

It has been shown that the induction window for acute cardioprotection is too short to permit synthesis of new protective proteins [26]. The up-regulation of existing defensive proteins via PTMs, which does not occur at the transcriptional level, likely accounts for the rapid protection. Earlier studies on SUR1, an isoform of SUR2, have shown that N-glycosylation can enhance SUR1 membrane targeting, expression level and channel complex stability [27,28]. To test whether SUR2 is modified by N-glycosylation, plasma membrane proteins were isolated from WT LV tissues; and de-glycosylated using peptide N-glycosidase F (PNGase F). We previously developed two SUR2-specific antibodies (Figure 4A); one was raised against an extracellular domain (T1) while the other was raised against an intracellular domain (BNJ-2). T1, but not BNJ-2, identified a putative N-glycosylation pattern in SUR2 in an earlier study [20]. BNJ-2 was then used as a negative control in this experiment. Both antibodies were employed to detect the SUR2 receptor species before and after the PNGase F treatment. When T1 was used to probe the samples, two bands at 150-kDa (complex-glycosylated) and 140-kDa (core-glycosylated) were detected in the untreated sample (Figure 4B). However, these two bands were reduced to a single 137-kDa non-glycosylated SUR2 in the PNGase F-treated sample, consistent with a prior report on the SUR1 de-glycosylation study [29]. The BNJ-2 antibody could not distinguish the different glycosylated SUR2 products as expected. Our data provided initial evidence that SUR2 is a glycoprotein. It is known that SUR1 has two alternative N-glycosylation sites [27-29]. An *in silico* analysis on SUR2 identified more than two putative N-glycosylation sites (N-X-S/T), which remain to be characterized.To understand whether SUR2 N-glycosylation level was altered in I-R, two additional protocols, a 90-min reperfusion (R) alone or a 30-min ischemia (I) alone, were included to dissect the timing and degree of PTM in treated hearts (Figure 4C). In this set of experiment, the R group was used as a reference control. When T1 was used to cross-react with the LV samples, both 150-kDa and 140-kDa SUR2 bands were detected (Figure 4D). The I-R or I group had a 2-fold or 1.5-fold significant increase (combined signals from both bands) in SUR2 expression relative to the R group.

**Figure 5:**
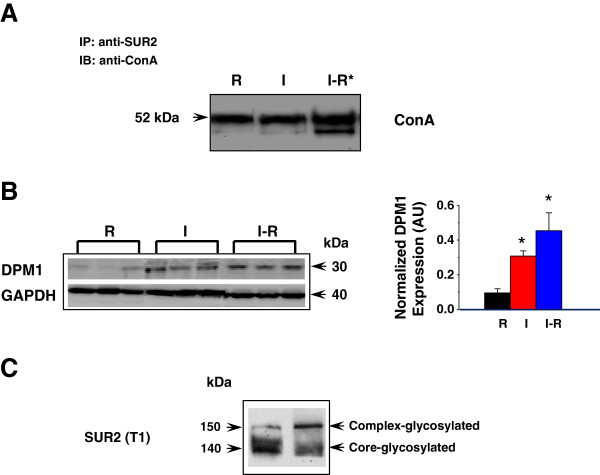
**Glycosylated SUR2 and estrogen regulation. (A)** Representative co- immunoprecipitation results in WT LV samples from R-, I- and IR- groups. The R group was used as a reference control. 500 μg protein (combined from 5 independently handled mice) was immuno-precipitated by 7 μg anti-SUR2 (T1), followed by immunoblotting using anti-concanavalin A (ConA) (1:1000). **(B)** Representative DPM1 Western blots in WT LV samples. Anti-DPM1 was used as the primary antibody (1:500). Secondary antibody was added at 1:10,000. n = 4. *, *p* < 0.05. The blot was stripped and re-probed with GAPDH (1:2000), and the density values were normalized to GAPDH. **(C)** Estrogen (E2) or DMSO (vehicle) treatment in cultured COS1 cells co-expressing Kir6.2/SUR2. T1 was used as the primary antibody (1:2000). Secondary antibody was added at 1:10,000.

Certain mannose-specific lectins, such as concanavalin A (ConA), can recognize N-linked glycans and bind to glycoproteins such as SUR1 [29]. We further tested whether SUR2 could interact with ConA in the three groups of hearts. Each sample was immunoprecipitated with T1 followed by immunoblotting using anti-ConA (Figure 5A). A ConA band in the size of 52-kDa was detected in all samples. A ConA monomer has a molecular weight of 26-kDa. The detected 52-kDa band may suggest that a dimeric form of ConA was associated with SUR2. We found that the I-R group had a 2.2-fold higher ConA level than R or I group. The results showed that SUR2 N-glycosylation level was increased in I-R hearts. A previous report has shown that the time required for transit/N-glycosylation of a (SUR1/Kir6.2)_4_ complex takes 2.2 h [30], which is very closed to the length of our I-R protocol. Our data (Figures 4D and 5A) therefore support the notion that N-glycosylation is likely the PTM that modifies SUR2 protein level in I-R hearts. The increased SUR2 level in the I group (Figure 4D), however, implies that other PTMs for SUR2 modifications may co-exist. A recent comparative glycoproteomic analysis in I-R or SHAM-treated male rat hearts (treated with PNGase F) reported that SUR2 glycosylation level was comparable in both groups [31]. This observation and our current study suggest that the up-regulation of SUR2 N-glycosylation may be female-specific.

Dolichol monophosphate mannose synthase (DPM1) catalyzes the first committed-step reaction in the N-glycosylation pathway [32]. DPM1 is responsible to transfer mannose from GDP-mannose to dolichol monophosphate to form dolichol monophosphate mannose as the mannosyl donor during N-glycosylation. Expression level of DPM1 was previously reported to be markedly enhanced by estrogen in mouse uteri [33]. In this experiment, we compared DPM1 levels in I-, R- and IR- treated mice. DPM1 level was increased by 4.5- or 3-fold in the I-R or I group relative to the R group (Figure 5B), indicating that the general capacity of N-glycosylation was also up-regulated in ischemic female hearts.

### SUR2 N-glycosylation is mediated by estrogen

Estrogen has been shown to increase membrane density of KATP channels in cultured H9c2 cells [34]. We tested whether E2 could further induce the glycosylated SUR2 receptor species using a heterologous expression system. A full-length SUR2 cDNA was introduced into a COS1 cell line that has a stably expressed Kir6.2 pore [20]. 24 h post transfection, cells were incubated with 100 nM E2 or DMSO (vehicle) for 24 h. When T1 was used to probe the samples, the core-glycosylated 140-kDa SUR2 was the major species in the DMSO-treated control cells. In the E2-treated cells, however, the complex-glycosylated 150-kDa SUR2 was the major species (Figure 5C). The data suggested that estrogen could further induce the complex-glycosylated SUR2. This species likely confers the glybenclamide-sensitive KATP currents in our earlier studies [19,20].

### SUR2KO Proteomic Changes Post I-R

**Figure 6:**
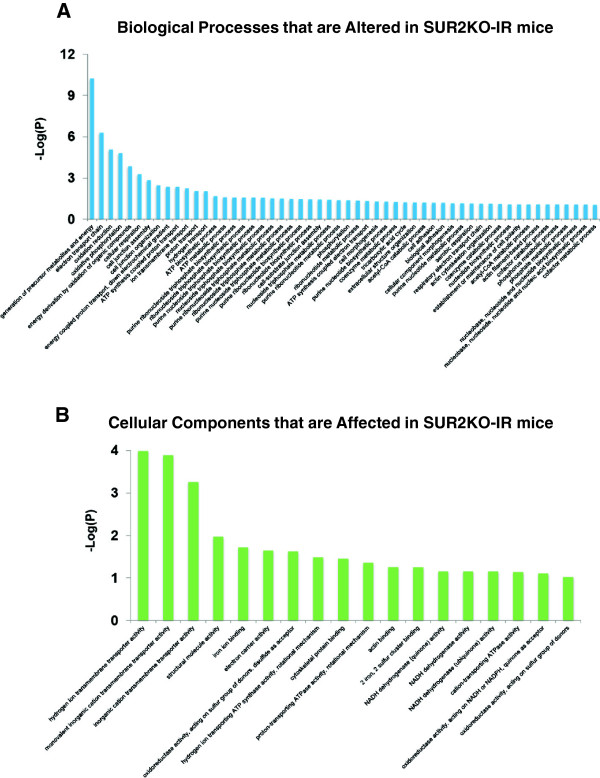
**Gene ontology enrichment analyses by DAVID using the 41 identified protein hits shown in Table **1**. (A)** Enriched biological processes categories. **(B)** Enriched cellular components categories. In **A-B**, *P*-values were used to report whether significance of overlaps with known functional categories was found. They were transformed into -Log values as shown on Y axis. Additional information is provided in Additional file 2.

**Figure 7:**
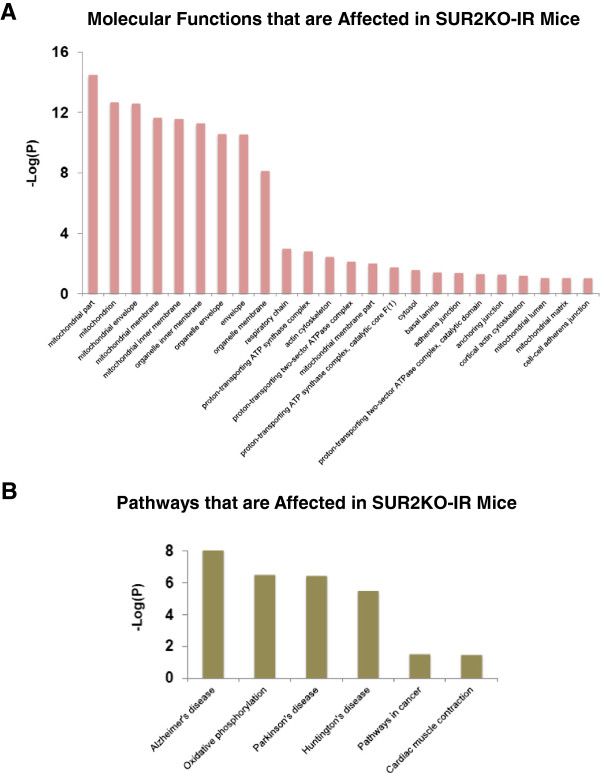
**Gene ontology and KEGG pathway enrichment analyses using the 41 identified protein hits shown in Table **1**. (A)** Enriched molecular function. **(B)** The 6 dysfunctional pathways identified in SUR2KO-IR mice. In **A**-**B**, *P*-values were used to report whether significance of overlaps with known functional categories was found. They were transformed into -Log values as shown on Y axis. Additional information is provided in Additional file 2.

KATP channels are recognized as metabolic sensors of the cell and they are indispensable in stress-induced adaptive response [35]. An earlier proteomic analysis in Kir6.2 knockout mice revealed a much stressed proteome under normal conditions [36]. These mice display larger infarcts and worse cardiac injury post I-R [37]. We expected that SUR2KO mice would be more complex because of the possible compensatory mechanisms conferred by multiple splice variants [38]. To explore pathway-level differences in KO-IR and WT-IR hearts, a comparative sub-proteomic profiling analysis was carried out. A spectral counting method was used to detect semi-quantitative changes between the two groups of mice. Purified samples prepared from three independently handled mice per group were subjected to mass spectrometry analysis. In the total 6 samples, we detected an average of 494 hits per sample. Subsequent annotation studies found that 41 proteins were differentially expressed in KO-IR and WT-IR hearts (Table 1). The DAVID functional annotation tool [39,40] was used to perform both Gene Ontology (GO) classifications and pathway enrichment analyses on the identified hits (Figures 6, 7 and Additional file 2). In the GO study, we identified 54 biological process categories (Figure 6A), 24 cellular component categories (Figure 6B) and 18 molecular function categories (Figure 7A). In the KEGG pathway enrichment study [41,42], we identified 6 major pathways that were altered in the KO mice (Figure 7B).

**Table 1:** **Protein hits that are differentially expressed in SUR2KO**-**IR and WT**-**IR hearts**

**Identified hits**	**Gene name**	***P-*Value**	**Fold of change (KO/WT)**
Mitochondrial peptide methionine sulfoxide reductase	Msra	0.0624981	0.19
1,4-alpha-glucan-branching enzyme	Gbe1	0.0221348	0.3
40S ribosomal protein S3	Rps3	0.0570084	0.32
Estradiol 17-beta-dehydrogenase 10	Hsd17b10	0.007576	0.48
Estradiol 17-beta-dehydrogenase 8	Hsd17b8	0.0257942	0.52
Dynamin-like 120 kDa protein	Opa1	0.0133927	0.59
Mitochondrial 2-oxoglutarate/malate carrier protein	Slc25a11	0.0229143	0.63
ATP synthase subunit gamma, mitochondrial	Atp5c1	0.0742584	0.66
ATP synthase subunit alpha, mitochondrial	Atp5a1	0.0866386	0.77
Cytochrome c oxidase subunit 5A, mitochondrial	Cox5a	0.0371111	0.79
Prohibitin-2	Phb2	0.0500592	1.3
Propionyl-CoA carboxylase beta chain, mitochondrial	Pccb	0.0813775	1.3
Heat shock protein HSP 90-beta	HSP90AB1	0.0215257	1.3
Cytochrome b-c1 complex subunit 1, mitochondrial	Uqcrc1	0.0602446	1.5
CDGSH iron-sulfur domain-containing protein 1	Cisd1	0.0446392	1.6
T-complex protein 1 subunit beta	Cct2	0.0880331	1.6
Basement membrane-specific heparan sulfate proteo-glycan core protein	Hspg2	0.0499444	1.6
Pyruvate dehydrogenase protein X component, mitochondrial	Pdhx	0.0121642	1.7
Catenin alpha-1	Ctnna1	0.0364573	1.7
NADH dehydrogenase [ubiquinone] 1 alpha subcomplex subunit 13	Ndufa13	0.0596356	1.7
Succinate dehydrogenase [ubiquinone] iron-sulfur subunit, mitochondrial	Sdhb	0.0079267	1.7
Electron transfer flavoprotein subunit beta	Etfb	0.0489129	1.8
ATP synthase subunit d, mitochondrial	Atp5h	0.0923448	1.8
Hemoglobin subunit beta-1	Hbb-b1	0.032562	1.8
Cytochrome b-c1 complex subunit 8	Uqcrq	0.0703449	1.9
Ferritin light chain 1	Ftl1	0.0305524	1.9
Cofilin-1	Cfl1	0.0619513	1.9
Acyl-CoA dehydrogenase family member 10	Acad10	0.0765324	2
NADH dehydrogenase [ubiquinone] 1 alpha subcomplex subunit 7	Ndufa7	0.089645	2
Adenosylhomocysteinase	Ahcy	0.0612594	2.1
Lipoprotein lipase	LPL	0.0690628	2.3
Talin-1	Tln1	0.0040294	2.3
Laminin subunit gamma-1	Lamc1	0.0178447	2.4
Junction plakoglobin	Jup	0.0020557	2.6
AFG3-like protein 2	Afg3l2	0.0210335	2.9
Glutathione S-transferase P 1	Gstp1	0.0086305	3
NSFL1 cofactor p47	Nsfl1c	0.0965357	3.3
Ubiquitin carboxyl-terminal hydrolase 14	Usp14	0.0142878	4.4
Vesicle-associated membrane protein-associated protein B	Vapb	0.0669842	4.9
Adenylyl cyclase-associated protein 1	Cap1	0.034702	5.6
Glutaredoxin-1	Glrx	0.0590062	Only detected in KO

Summary data of semi-quantitative proteomic comparisons using SUR2KO-IR and WT-IR hearts. *P*-values were calculated using a pair-wise *t*-test based on three replicates from each group of mice. *p* < 0.1 was considered statistically significant. The fold of expression change is shown as a ratio of KO/WT. The cut-off is set at 1.3-fold of difference.

Our physiological data (Figures 2 and 3) showed that SUR2KO female mice resembled those aged WT females in prior reports [2-4], which display a compromised estrogenic effect and cardioprotection. In our proteomic study, levels of estradiol 17β-dehydrogenase 8 (Hsd17β8) and 10 (Hsd17β10) were found to be significantly decreased by 2-folds in KO-IR mice. Hsd17β-based enzymes belong to the short-chain dehydrogenase/reductase superfamily. They are mainly involved in the biosynthesis of estrogens, androgens and fatty acids [43]. Hsd17β8 catalyzes the inter-conversion between E2 (estradiol) and E1 (estrone). E2 is the predominant form of circulating estrogen before menopause while E1 is the major estrogen type present in the postmenopausal stage [44]. Hsd17β8 primarily acts as an oxidative enzyme to inactivate E2. However, it has some reductase activity and can produce E2 from E1. Thus, Hsd17β8 is an important Hsd17β member that controls concentration of estrogen in the cell. It seems that KO-IR female mice are “locked” into a relatively high E1 state, mimic the postmenopausal women group. The high E1 level may auto-suppress the activity of Hsd17β8, which prevents further inactivation of E2 or over-accumulation of E1. Our results therefore provided first line of evidence that disrupting KATP channels affects estrogen biosynthesis in mice. The loss of KATP as a downstream target for E2 modulation likely affects other related molecular signaling pathways (Additional file 2). On the other hand, Hsd17β10 mainly functions in the mitochondria to catalyze beta-oxidation at position 17 of estrogen or androgen. An earlier study has reported that Hsd17β10 deficiency caused by genetic mutations can result in Alzheimer’s disease [45]. In addition to the findings in estrogen-related targets, we found hits (Lamc1, Hspg2 and LPL) that are related to N-glycosylation in our GO study. Levels of these three proteins were found to be higher in the KO-IR hearts. A previous study showed that level of N-glycosylation in Lamc1 or Hspg2 was altered in I-R rat hearts [31]. Changes in these proteins could be related to the increase of general N-glycosylation capacity in I-R. We also noticed that levels of certain targets related to actin cytoskeleton were altered. The alteration may be associated with the detected hypertrophy in KO hearts.

More recent studies have shown that KATP channels are also present in the inner membranes of mitochondria [46]. We previously reported that the diazoxide-sensitive mitochondrial KATP activity was absent in SUR2KO mice [21]. In our proteomic study, we found that 50% of the identified protein hits are associated with mitochondrial function and energy generation (Table 1). The finding revealed that the KO female mitochondria were severely altered in I-R. One important hit, optic atrophy 1 (OPA1), is known to play a key role in shaping the mitochondria and fusion to the inner membrane [47]. A recent report shows that a decreased level of OPA1 is associated with heart failure [48]. We detected a significant 2-fold reduction in OPA1 level in SUR2KO-IR hearts, suggesting that disrupting KATP likely affects OPA1 expression and the fusion/fission rate of mitochondria. It is known that estrogen in involved in modulating mitochondrial biogenesis and maintaining mitochondrial membrane potential [3]. Functional estrogen receptor, ERβ, has been found in the mitochondria [49] to mediate anti-apoptotic response [50] and cardioprotection [8,25]. The large numbers of mitochondrial hits identified in our proteomic study may be due to a combined effect from losing the diazoxide-sensitive mitochondrial KATP activity and altered mitochondrial ERβ level. Future compartment-specific proteomic analyses using enriched fractions isolated from mitochondrial or plasma membranes may identify additional targets that are related to estrogen regulation and female-specific stress signaling network.

## Conclusions

The regulatory subunit for the cardiac KATP channels, SUR2, is a low-affinity sulfonylurea receptor [28]. Because sulfonylureas are commonly used in treating diabetes mellitus, knowledge about SUR2 regulation by estrogen is expected to shed new light into hormonal regulation of a “cardioprotective” ion channel and the related molecular signaling pathways. Modulating activities of key ion channels via targeting their regulatory subunits has been employed as a new therapeutic strategy to treat certain cardiovascular diseases. Increasing SUR2 expression by N-glycosylation mediated by estrogen may be an effective manner to enhance KATP channel density in the heart. The novel finding that KATP is a downstream regulatory target of estrogen will provide new perspectives in future estrogen replacement therapies to postmenopausal women. The different I-R response in both genders of SUR2KO mice indicates that these models are innovative platforms to study gender-specific divergence in other cardiovascular diseases.

## Methods

### Mice

Mouse protocols and handling were performed following the guidelines of National Institutes of Health. All mice were maintained at the University of Wisconsin Animal Core Facility. SUR2 knockout mice were previously created by inserting a disruption cassette at exons 12–16 of the SUR2 gene [19]. C57BL-6 J mice (Jackson Laboratories, Bar Harbor, Maine) that were heterozygous for the *Sur2* locus were bred into the FVB background [51]. Heterologous mice were interbred and genotyped to obtain homozygous mutants. Age-matched female SUR2KO and WT littermates were used in this study.

### Related antibodies

SUR2 antibodies were custom-designed antibodies that were generated as previously described [20]. Anti-GAPDH was obtained from Assay Designs (Ann Arbor, MI); anti-ERα was from Santa Cruz; anti-ERβ was from Millipore; anti-concanavalin A was from Vector Laboratories (Burlingame, CA) and anti-DPM1 was from IMGENEX (San Diego, CA). Secondary antibodies were obtained from GE Healthcare (Piscataway, NJ).

### Echocardiography

Mice were lightly anesthetized before echocardiographic recordings using Vevo Model770 (VisualSonics, Canada). Isoflurane was delivered at 3% during induction and at 1% for maintenance via nose cone. Images were captured using a 40-mHz mechanical transducer.

### Ischemia-reperfusion procedure

An ischemia-reperfusion (I-R) protocol operated as previously described with modifications [22,23]. An open-chest occlusion model was used to induce I-R in SUR2KO and WT female mice. Mice were induced with 2% isoflurane, intubated and ventilated at 150 breaths/min at 200–300 μL tidal volume. Body temperature was maintained with a heating pad. Electro-cardiogram (ECG) was used to monitor mice and the ECG lead was processed with a Gould amplifier and digitally converted for off-line analysis. The protocol included a 30-min ischemia phase followed by a 90-min reperfusion period. Epicardial cyanosis, alteration in myocardial contractility, and ST segment elevation were used to confirm ischemia. Reperfusion was initiated by unclamping the hemostat and loosening the suture from the polyethylene tubing, and it was confirmed by elimination of epicardial cyanosis and normalization of the S-T segment. Infarct sizes were determined as previously described [52].

### Estrogen delivery in mice

Adult WT or SUR2KO female mice at the age of 9 wks were subject to ovariectomization [25]. These mice were subsequently implanted with 17β-estradiol (E2) or placebo pellets (0.1 μg/g/day, 21-day release, Innovative Research of America, Sarasota, FL). All mice received our I-R protocol on Day 18-20.

### Quantitative RT-PCR

Total RNA was isolated from LV tissues using TRIzol reagents (Life Technologies) and RT-PCR reactions were carried out as previously described [25]. Primers used to amplify SUR2A are: 5’-TGGTGGTACCTCACTTCAGGA-3’ and 5’-CAGGATGGTTTATACTGTA- TTCGGA-3’. Controls primers used to amplify GAPDH are: 5’-AGACATCTAAGGTT- CCAGTATGAC-3’ and 5’-ATCGTCCCATTTGATGTTAGAG-3’. Banding density was scanned by the UVP BioSpectrum Imaging System (Upland, CA) and normalized to GAPDH.

### Protein extraction and Western blot analysis

LV tissues were carefully isolated for protein extraction and concentrations were determined using a DC Protein Assay Kit (Bio-Rad, Hercules, CA) as previously reported. Protein samples were separated on 4-12% MOPS NuPAGE gels unless stated elsewhere. Western blots were performed more than three times. Chemiluminescence was detected using an ECL-Plus Detection Kit (GE Healthcare). Blots were scanned and banding densities were determined by the UVP Imaging System.

### De-glycosylation Treatment

Mouse LV tissues were isolated from 10–12 wks-old female hearts and subjected to membrane protein isolation. Plasma membrane proteins were purified from mouse LV or cell lysates using a MEM-PER Eukaryotic Membrane Protein Extraction kit (Thermo). 300 μg LV membrane proteins were treated by an enzymatic de-glycosylation reaction using a peptide N-glycosidase F (PNGase F) following manufacturer’s protocol (Sigma, St. Louis, MO). Protein samples were ran on 3-8% Tris-acetate NuPAGE gels to allow better separation. The blots were probed with anti-SUR2 (T1) at 1:2000 or BNJ-2 at (1:1000).

### Estrogen treatment in cultured cells

For COS1 cell culture, cells were seeded on a 35-mm-diameter plate (1×10^5^) containing complete MEM medium (Life Technologies), 10% fetal bovine serum, 2 mM L-glutamine, 0.1 nM MEM non-essential amino acid solution, 1 mM MEM pyruvate solution, 10 U penicillin and 10 g streptomycin. SUR2A was co-transfected into a COS1 line stably expressed a Kir6.2 pore [20] using a TransIT-COS transfection kit (Mirus, Madison WI). 24 h post transfection, cells were treated with 100 nM E2 or DMSO (vehicle) for 24 h. Cells were subjected to membrane protein isolation for Western blot analysis.

### Co-Immunoprecipitation (Co-IP)

Co-IP experiments were carried out using a Classic Co-IP Kit (Thermo) following manufacturer’s recommended procedures. 7 μg anti-SUR2 (T1) was used to IP 500 μg LV proteins (combined from five independently handled mice) from R-, I- or IR- mice followed by immunoblotting using an anti-ConA.

### Proteomic study

Isolated LV tissues from three independently handled SUR2KO-IR or WT-IR mice were used for protein extraction in the presence of one protease cocktail inhibitor tablet (Roche, Indianapolis, IN). 600 μg protein from each sample was subjected to an albumin depletion column (Qiagen) to remove excessive albumin. “In-liquid” digestion and subsequent mass spectrometric analysis were carried out at the University of Wisconsin Mass Spectrometry Facility. 200 μg of albumin-depleted protein sample was re-solubilized and denatured in 15 μL buffer containing 8 M urea, 50 mM NH_4_HCO_3_ (pH8.5) and 1 mM Tris–HCl (pH7.5) for 10 min at room temperature. The mixture was diluted to 60 μL in the reduction/ alkylation step by adding 2.5 μL of 25 mM DTT, 5 μL MeOH and 37.5 μL of 25 mM NH_4_HCO_3_ (pH8.5). The mixture was incubated at 50°C for 15 min. Once it was cooled down to room temperature, 3 μL of 55 mM iodoacetamide was added and the mixture was incubated in the dark at room temperature for 15 min. The reaction was quenched by adding 8 μL of 25 mM DTT. Peptides were released using 30 μL Trypsin Gold solution (100 ng/μL in 25 mM NH_4_HCO_3_, Promega, Madison, WI) to reach a 100-μL final volume. Digestion was conducted at 42°C for 1 h. Then 15 μL of fresh trypsin solution was added to reach a final enzyme:substrate ratio of 1 to 44, and the digestion reaction was carried out at 37°C for overnight. The reaction was terminated by adding 2.5% trifluoroacetic acid to reach a 0.3% final concentration. 8 μL (12 μg) of the peptide mixture was loaded for subsequent mass spectrometry analysis.

### Mass spectrometry procedure

Peptides were analyzed by Nano-LC-MS/MS using the Agilent 1100 NanoFlow System (Agilent Technologies, Santa Clara, CA), which was connected to a hybrid Linear Ion Trap-OrbiTrap Mass Spectrometer (LTQ-OrbiTrap [53]) equipped with a Nano-electrospray ion source (Thermo-Fisher Scientific). HPLC was performed using a 15-cm column packed with MAGIC C18AQ 3-μm 200 Å particles (MICHROM BioResources, Auburn, CA) and a P-2000 laser pulled tip (Sutter Instrument, Novato, CA) that was connected with a 360 μm × 75 μm fused silica tubing. Sample loading and desalting were carried out at a 10 μL/min rate using a Zorbax 300SB-C18 trapping column (Agilent Technologies), which was in line with an auto-sampler. Peptide elution was carried out using solvents comprised of 0.1% formic acid in water (Solvent A) and 0.1% formic acid/95% acetonitrile in water (Solvent B). The gradient consisted of a 20-min loading and desalting period with a stepwise column equilibration at 1% Solvent B initially, ramp to 40% B for 195 min, ramp to 60% B for 20 min, ramp to 100% B for 5 min and held for 3 min. The column was then re-equilibrated at 1% Solvent B for 30 min. The flow rate for peptide elution and re-equilibration was 0.2 μL/min.

### Spectra collection

The LTQ-Orbitrap was set to acquire MS/MS spectra in a data-dependent mode. MS survey scans from m/z 300 to 2000 were collected in centroid mode at a resolving power of 100,000. In each scan, MS/MS spectra were collected from the five most-abundant output signals. Dynamic exclusion was employed to increase dynamic range and maximize peptide identification. This feature excluded precursors up to 0.55 m/z below and 1.05 m/z above those previously selected precursors. Precursors remained on the exclusion list for 40 s. Singly-charged ions or ions for which the charge state could not be assigned were rejected for further consideration.

### Database analysis

Raw MS/MS data was searched against the UniProt Mouse Amino Acid Sequence Database, which contains over 30,634 protein entries. A Mascot Search Engine 2.2.07 (Matrix Science, Boston, MA) was used in the search with fixed cysteine carbamidomethylation, variable methionine oxidation and asparagine/glutamine deamidation. Peptide mass tolerance was set at 20 ppm and fragment mass at 0.8 Dalton. Protein annotation, significance of identification and spectral based quantification were carried out using the Scaffold Software Package 4.1.1 (Proteome Software, Portland, OR). In this version, quantitative values of protein hits were normalized to total spectral counts, enhancing the accuracy in target identification. Samples from three independently handled KO-IR or WT-IR mice were analyzed. Protein probabilities were assigned by the Protein Prophet algorithm [54]. An average of 494 hits was identified in each sample. Protein identifications were accepted if they could be established at greater than 95.0% probability and contained at least two identified peptides. Among the total 612 protein hits found from the three sets of samples, the prophet false discovery rate (FDR) for proteins was at 0.1%. Peptide identifications were accepted if they could be established at greater than 90.0% probability as specified by the Peptide Prophet algorithm [55]. Based on the ~66,000 spectra that were found from the three sets of samples, the prophet FDR for spectra was at 0.9%. Proteins that contained similar peptides and could not be differentiated based on MS/MS analysis alone were grouped to satisfy the principles of parsimony.

### Pathway enrichment analysis

Using the normalized spectrum data collected from KO-IR or WT-IR mice, Gene Ontology [39,40] and pathway enrichment [41,42] were performed using the DAVID (Database for Annotation, Visualization and Integrated Discovery) Bioinformatics Resources v6.7 (NIAID, NIH). DAVID categorizes genes based on enrichment of GO classification, Kyoto Encyclopedia of Genes and Genomes (KEGG), and information from other databases. *P*-values were used to report whether significance of overlaps with known functional categories was found.

### Other statistical analysis

Data were reported as mean ± SEM in the echocardiography and I-R experiments while other data were reported as mean ± STDEV. Statistical analysis was performed using Origin Version9. Statistical significance was determined by Student *t* test (2-tailed) for two experimental groups or one-way ANOVA for multiple groups with post hoc test using the Bonferroni method. A *p* < 0.05 was considered statistically significant.

## Competing interest

The authors declare that they have no competing interests.

## Authors’ contributions

JG and DX performed proteomic data annotations, data interpretation, pathway enrichment and writing of manuscript sections. GS performed sample preparations, mass spec runs, spectra collecting and writing of manuscript sections. HV provided technical consultation and edited the manuscript. WX provided tools and technical consultation and edited the manuscript. NQS conceptualized the study, designed experiments, interpreted data, wrote the manuscript and obtained the funding. All authors read and approved the final manuscript.

## Supplementary Material

Additional file 1: Figure S1 **(A)**. Average area-at-risk over left ventricular region ratios recorded in SUR2KO and WT hearts post I-R. Treated mice had similar area-at-risk over LV ratios post I-R, n=6-8. **(B)**. Ovariectomized mice implanted with either estrogen (E2) or placebo pellets displayed comparable area-at-risk over LV ratios after I-R treatment. n=4-5. This is a control parameter that shows our consistency in surgical handling. Click here for file

Additional file 2: Table S1 Biological Process GO Classification. **Table S2 and Table S3.** Molecular Function GO Classification. **Table S4.** Affected pathways. Click here for file

## References

[B1] Murry CE Jennings RB Reimer KA Preconditioning with ischemia: A delay of lethal cell injury in ischemic myocardium Circulation 1986 11 1124 1136 10.1161/01.CIR.74.5.1124 3769170

[B2] Hayward CS Kelly RP Collins P The roles of gender, the menopause and hormone replacement on cardiovascular function Cardiovasc Res 2000 11 28 49 10.1016/S0008-6363(00)00005-5 10727651

[B3] Murphy M Steenbergen C Gender-based differences in mechanisms of protection in myocardial ischemia-reperfusion injury Cardiovas Res 2007 11 478 486 10.1016/j.cardiores.2007.03.025 17466956

[B4] Lioyd-Jones D Adams RJ Brown TM Carnethon M Dai SF De Simone G Ferguson TB Ford E Furie K Gillespie C Go A Greenlund K Haase N Hailpern S Ho PM Howard V Kissela B Kittner S Lackland D Lisabeth L Marelli A McDermott MM Meigs J Mozaffarian D Mussolino M Nichol G Roger VL Rosamond W Sacco R Sorlie P Roger VL Thom T Wasserthiel-Smoller S Wong ND Wylie-Rosett J Heart disease and stroke statistics 2010 update Circulation 2010 11 e46 e238 20019324 10.1161/CIRCULATIONAHA.109.192667

[B5] Matthews J Gustafsson JA Estrogen signaling: A subtle balance between ERα and ERβ Mol Interv 2003 11 281 292 10.1124/mi.3.5.281 14993442

[B6] Couse JF Lindzey J Grandien K Gustafsson JA Korach KS Tissue distribution and quantitative analysis of estrogen receptor α and estrogen receptor β messenger ribonucleic acid in the wild-type and ERα knockout mouse Endocrinol 1997 11 4613 4621 10.1210/endo.138.11.5496 9348186

[B7] Lizotte E Grandy SA Tremblay A Expression, distribution and regulation of sex steroid hormone receptors in mouse heart Cell Physiol Biochem 2009 11 75 86 10.1159/000204096 19255502

[B8] Gabel SA Walker VR London RE Steenbergen C Korach KS Murphy E Estrogen receptor beta mediates gender differences in ischemia/reperfusion injury J Mol Cell Cardiol 2005 11 289 297 10.1016/j.yjmcc.2004.11.013 15698835

[B9] Wang M Crisostomo P Wairiuko GM Meldrum DR Estrogen receptor-alpha mediates acute myocardial protection in females Am J Physiol Heart Circ Physiol 2006 11 H2204 2209 10.1152/ajpheart.01219.2005 16415070

[B10] Noma A ATP-regulated K + channels in cardiac muscle Nature 1983 11 147 148 10.1038/305147a0 6310409

[B11] Gross GJ Auchampach JA Blockade of ATP-sensitive potassium channels prevents myocardial preconditioning in dogs Circ Res 1992 11 223 233 10.1161/01.RES.70.2.223 1310443

[B12] Isomoto S Kondo C Yamada M Matsumoto S Higashiguchi O Horio Y Matsuzawa Y Kurachi Y A novel sulfonylurea receptor forms with BIR (Kir6.2) a smooth muscle type ATP-sensitive K+ channel J Biol Chem 1986 11 24321 24324 10.1074/jbc.271.40.24321 8798681

[B13] Inagaki N Gonoi T Clement JP Wang CZ Aguilar-Bryan L Bryan J Seino S A family of sulfonylurea receptors determines the pharmacological properties of ATP-sensitive K + channels Neuron 1996 11 1011 1017 10.1016/S0896-6273(00)80124-5 8630239

[B14] Chutkow WA Simon MC Le Beau MM Burant CF Cloning, tissue expression, and chromosomal localization of SUR2, the putative drug-binding subunit of cardiac, skeletal muscle, and vascular KATP channels Diabetes 1996 11 1439 1445 10.2337/diab.45.10.1439 8826984

[B15] Clement JP Kunjilwar K Gonzalez G Schwanstecher M Panten U Aguilar-Bryan L Bryan J Association and stoichiometry of K(ATP) channel subunits Neuron 1997 11 827 838 10.1016/S0896-6273(00)80321-9 9182806

[B16] Ranki HJ Budas GR Crawford RM Jovanovic A Gender-specific difference in cardiac ATP-sensitive K channels J Am Col Cardiol 2001 11 906 915 10.1016/S0735-1097(01)01428-0 11527652

[B17] Johnson MS Moore RL Brown DA Sex differences in myocardial infarct sizes are abolished by sarcolemmal KATP channel blockade in rat Am J Physiol Heart Circ Physiol 2006 11 H2644 2647 10.1152/ajpheart.01291.2005 16473955

[B18] Chen F Wetzel GT Friedman WF Klitzner TS ATP-sensitive potassium channels in neonatal and adult rabbit ventricular myocytes Pediatr Res 1992 11 230 235 10.1203/00006450-199208000-00021 1508616

[B19] Chutkow WA Samuel V Hansen PA Pu JL Valdivia CR Makielski JC Burant CF Disruption of Sur2-containing KATP channels enhances insulin-stimulated glucose uptake in skeletal muscle Proc Natl Acad Sci USA 2001 11 11760 11764 10.1073/pnas.201390398 11562480 PMC58803

[B20] Pu JL Ye B Kroboth SL McNally EM Makielski JC Shi NQ Cardiac sulfonylurea receptor short form-based channels confer a glibenclamide-insensitive KATP activity J Mol Cell Cardiol 2008 11 188 200 10.1016/j.yjmcc.2007.09.010 18001767 PMC2276634

[B21] Ye B Kroboth SL Pu JL Sims JJ Aggarwal NT McNally EM Makielski JC Shi NQ Molecular identification and functional characterization of a mitochondrial SUR2 variant generated by intra-exonic splicing Circ Res 2009 11 1083 1093 10.1161/CIRCRESAHA.109.195040 19797704 PMC2988690

[B22] Kukielka GL Youker KA Michael LH Kumar AG Ballantyne CM Smith CW Entman ML Role of early reperfusion in the induction of adhesion molecules and cytokines in previously ischemic myocardium Mol Cell Biochem 1995 11 5 12 10.1007/BF00944777 7494554

[B23] Guo Y Wu WJ Qiu Y Tang XL Yang Z Bolli R Demonstration of an early and a late phase of ischemic preconditioning in mice Am J Physiol Heart Circ Physiol 1998 11 H1375 1387 10.1152/ajpheart.1998.275.4.H1375 PMC3701297 9746488

[B24] Stoller D Kakkar R Smelley M Chalupsky K Earley JU Shi NQ Makielski JC McNally EM Mice lacking sulfonylurea receptor 2 ATP sensitive potassium channels are resistant to acute cardiovascular stress J Mol Cell Cardiol 2007 11 445 454 10.1016/j.yjmcc.2007.07.058 17765261 PMC2745323

[B25] Lin J Steenbergen C Murphy E Sun J Estrogen receptor-beta activation results in S-nitrosylation of proteins involved in cardioprotection Circulation 2009 11 245 254 10.1161/CIRCULATIONAHA.109.868729 19581491 PMC2778304

[B26] Thornton J Striplin S Liu GS Swafford A Stanley AWH Van Winkle DM Downey JM Inhibition of protein synthesis does not block myocardial protection afforded by preconditioning Am J Physiol Heart Circ Physiol 1990 11 H1822 1825 10.1152/ajpheart.1990.259.6.H1822 2260706

[B27] Conti LR Radeke CM Shyng S-L Vandenberg CA Transmembrane topology of the sulfonylurea receptor SUR1 J Biol Chem 2001 11 41270 41276 10.1074/jbc.M106555200 11546780

[B28] Aguilar-Bryan L Clement JP Gonzalez G Kunjilwar K Babenko A Bryan J Toward understanding the assembly and structure of KATP channels Physiol Rev 1998 11 227 245 9457174 10.1152/physrev.1998.78.1.227

[B29] Nelson DA Bryan J Wechsler S Clement JP Aguilar-Bryan L The high-affinity SUR: distribution, glycosylation, purification and immunoprecipitation of two forms from endocrine and neuroendocine cell lines Biochem 1996 11 14793 14799 10.1021/bi960777y 8942641

[B30] Crane A Aguilar-Bryan L Assembly, maturation, and turnover of KATP channel subunits J Biol Chem 2004 11 9080 9090 10.1074/jbc.M311079200 14699091

[B31] Parker BL Palmisano G Edwards AVG White MY Engholm-Keller K Lee A Scott NE Kolarich D Hambly BD Packer NH Larsen MR Stuart J Cordwell SJ Quantitative *N*-linked glycoproteomics of myocardial ischemia and reperfusion injury reveals early remodeling in the extracellular environment Mol Cell Proteomics 2011 11 1 13 10.1074/mcp.M110.006833 PMC3149091 21441315

[B32] Ohtsubo K Marth JD Glycosylation in cellular mechanisms of health and disease Cell 2006 11 855 867 10.1016/j.cell.2006.08.019 16959566

[B33] Carson DD Farrar JD Laidlaw J Wright DA Selective activation of the N-glycosylation apparatus in uteri by estrogen J Biol Chem 1990 11 2947 2955 2303433

[B34] Ranki HJ Budas GR Crawford RM Davies AM Jovanovic A 17β-Estradiol regulates expression of KATP channels in heart-derived H9c2 cells J Am Col Cardiol 2002 11 367 374 10.1016/S0735-1097(02)01947-2 12106946

[B35] Hodgson DM Zingman LV Kane GD Perez-Terzic C Bienengraeber M Ozcan C Gumina RJ Pucar D O'Coclain F Mann DL Alekseev AE Terzic A Cellular remodeling in heart failure disrupts KATP channel-dependent stress tolerance EMBO J 2003 11 1732 1742 10.1093/emboj/cdg192 12682006 PMC154482

[B36] Arrell DK Zlatkovic J Kane GC Yamada S Terzic A ATP-sensitive K channel knockout induces cardiac proteome remodeling predictive of heart disease susceptibility J Proteome Res 2009 11 4823 4834 10.1021/pr900561g 19673485 PMC2818626

[B37] Suzuki M Sasaki N Miki T Sakamoto N Ohmoto-Sekine Y Tamagawa M Seino S Marban E Nakaya H Role of sarcolemmal K(ATP) channels in cardioprotection against ischemia/reperfusion injury in mice J Clin Invest 2002 11 509 516 10.1172/JCI0214270 11854323 PMC150878

[B38] Shi NQ Ye B Makielski JC Function and distribution of the SUR isoforms and splice variants J Mol Cell Cardiol 2005 11 51 60 10.1016/j.yjmcc.2004.11.024 15978902

[B39] Huang DW Sherman BT Lempicki RA Systematic and integrative analysis of large gene lists using DAVID bioinformatics resources Nat Protocols 2008 11 44 57 10.1038/nprot.2008.211 19131956

[B40] Huang DW Sherman BT Lempicki RA Bioinformatics enrichment tools: paths toward the comprehensive functional analysis of large gene lists Nucleic Acids Res 2009 11 1 13 10.1093/nar/gkn923 19033363 PMC2615629

[B41] Nakao M Bono H Kawashima S Kamiya T Sato K Goto S Kanehisa M Genome-scale gene expression analysis and pathway reconstruction in KEGG Genome Inform 1999 11 94 103 11072346

[B42] Kanehisa M KEGG for integration and interpretation of large-scale molecular data sets Nucleic Acids Res 2012 11 D109 D114 10.1093/nar/gkr988 22080510 PMC3245020

[B43] Marchais-Oberwinkler S Henn C Möller G Klein T Negri M Oster A Spadaro A Werth R Wetzel M Xu K Frotscher M Hartmann RW Adamski J 17β-Hydroxysteroid dehydrogenases (17β-HSDs) as therapeutic targets: protein structures, functions, and recent progress in inhibitor development J Steroid Biochem Mol Biol 2011 11 66 82 10.1016/j.jsbmb.2010.12.013 21193039

[B44] Rotinen M Celay J Alonso MM Arrazola A Encio I Villar J Estradiol induces type 8 17beta-hydroxysteroid dehydrogenase expression: crosstalk between estrogen receptor alpha and C/EBPbeta J Endocrinol 2009 11 85 92 10.1677/JOE-08-0134 18852215

[B45] Yan SD Fu J Soto C Chen X Zhu H Al-Mohanna F Collison K Zhu A Stern E Saido T Tohyama M Ogawa S Roher A Stern D An intracellular protein that binds amyloid-beta peptide and mediates neurotoxicity in Alzheimer's disease Nature 1997 11 689 695 10.1038/39522 9338779

[B46] Inoue I Nagase H Kishi K Higuti T ATP-sensitive K + channel in the mitochondrial inner membrane Nature 1991 11 244 247 10.1038/352244a0 1857420

[B47] Chen L Gong Q Stice JP Knowlton AA Mitochondrial OPA1, apoptosis, and heart failure Cardiovasc Res 2009 11 91 99 10.1093/cvr/cvp181 19493956 PMC2741347

[B48] Chen L Liu T Tran A Lu X Tomilov AA Davies V Cortopassi G Chiamvimonvat N Bers DM Votruba M Knowlton AA OPA1 mutation and late-onset cardiomyopathy: mitochondrial dysfunction and mtDNA instability J AM Heart Asso 2012 11 5 003012 10.1161/JAHA.112.003012 PMC3541627 23316298

[B49] Yang SH Liu R Perez EJ Wen Y Stevens SM Jr Valencia T Mitochondrial localization of estrogen receptor beta Proc Natl Acad Sci USA 2004 11 4130 4135 10.1073/pnas.0306948101 15024130 PMC384706

[B50] Parkash J Felty Q Roy D Estrogen exerts a spatial and temporal influence on reactive oxygen species generation that precedes calcium uptake in high-capacity mitochondria: implications for rapid nongenomic signaling of cell growth Biochem 2006 11 2872 2881 10.1021/bi051855x 16503642

[B51] Kakkar R Ye B Stoller DA Smelley M Shi NQ Galles K Hadhazy M Makielski JC McNally EM Spontaneous coronary vasospasm in KATP mutant mice arises from a smooth muscle-extrinsic process Circ Res 2006 11 675 689 10.1161/01.RES.0000209516.84815.3e 16456098

[B52] Ytrehus K Liu Y Tsuchida A Miura T Liu GS Yang X Herbert D Cohen MV Downey JM Rat and rabbit heart infarction: effects of anesthesia, perfusate, risk zone, and method of infarct sizing Am J Physiol 1994 11 H2383 H2390 7528994 10.1152/ajpheart.1994.267.6.H2383

[B53] Haqqani AS Kelly JF Stanimirovic DB Quantitative protein profiling by mass spectrometry using label-free proteomics Methods Mol Biol 2008 11 241 256 10.1007/978-1-59745-188-8_17 18370108

[B54] Nesvizhskii AI Keller A Kolker E Aebersold R A statistical model for identifying proteins by tandem mass spectrometry Anal Chem 2003 11 4646 4658 10.1021/ac0341261 14632076

[B55] Keller A Nesvizhskii AI Kolker E Aebersold R Empirical statistical model to estimate the accuracy of peptide identifications made by MS/MS and database search Anal Chem 2002 11 5383 5392 10.1021/ac025747h 12403597

