# Supplementary material for: Disrupting KATP channels diminishes the estrogen-mediated protection in female mutant mice during ischemia-reperfusion
Source: Clin Proteomics. 2014 May 6;11(1):19. doi: 10.1186/1559-0275-11-19 (PMC4047774; doi:10.1186/1559-0275-11-19)
Supplement: Additional file 1: Figure S1 — (A). Average area-at-risk over left ventricular region ratios recorded in SUR2KO and WT hearts post I-R. Treated mice had similar area-at-risk over LV ratios post I-R, n=6-8. (B). Ovariectomized mice implanted with either estrogen (E2) or placebo pellets displayed comparable area-at-risk over LV ratios after I-R treatment. n=4-5. This is a control parameter that shows our consistency in surgical handling. [file 1559-0275-11-19-S1.pdf]

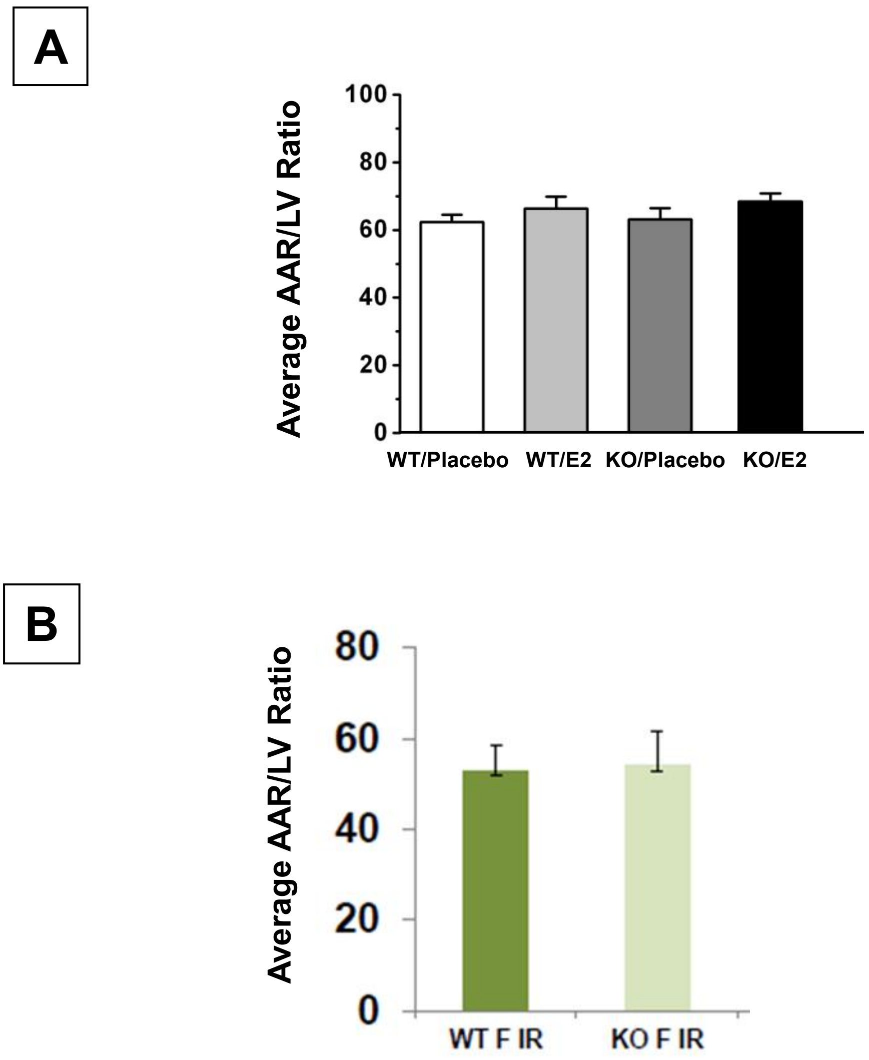

Figure S1. (**A**) Average area-at-risk over left ventricular region ratios recorded in SUR2KO and WT hearts post I-R. Treated mice had similar area-at-risk over LV ratios post I-R, n=6-8.
(**B**) Ovariectomized mice implanted with either estrogen (E2) or placebo pellets displayed comparable area-at-risk over LV ratios after I-R treatment. n=4-5. This is a control parameter that shows our consistency in surgical handling.
